# GWAS studies and polygenic risk scores in organ transplantation

**DOI:** 10.1097/MOT.0000000000001316

**Published:** 2026-09-25

**Authors:** Sabrina Stimmeder, Eleonora A.M. Festen, Vincent E. de Meijer

**Affiliations:** a Department of Surgery, Section of Hepatobiliary Surgery and Liver Transplantation, University Medical Center Groningen and University of Groningen, Groningen, The Netherlands; b Department of Surgery, Division of General, Visceral and Transplant Surgery, Medical University of Graz, Graz, Austria; c Department of Gastroenterology and Hepatology, University Medical Center Groningen and University of Groningen; d UMCG Comprehensive Transplant Center, University Medical Center Groningen, Groningen, The Netherlands

**Keywords:** genetic association study, genetic risk score, genome-wide association study, organ transplantation, polygenic risk score

## Abstract

**Purpose of review:**

To summarize recent developments published between 1 January 2024 and 15 August 2026 in genome-wide and polygenic analyses of solid organ transplantation, with a focus on kidney, heart, liver, and lung transplantation.

**Recent findings:**

Recent studies demonstrate that posttransplant phenotypes can reflect distinct genetic contributions from the recipient, donor organ, and donor-recipient pair. Donor and recipient polygenic risk scores have been associated with graft function, graft survival, and posttransplant complications, although their incremental predictive value beyond clinical factors has generally been modest. Genome-wide analyses of donor-recipient mismatch have identified genetic differences outside the HLA region associated with rejection and graft loss. Emerging evidence suggests that the biological relevance of mismatch depends not only on the extent of genetic difference, but also on its functional nature and direction. However, most studies remain limited by small, highly selected cohorts, limited independent replication, and predominant European ancestry.

**Summary:**

Genome-wide and polygenic approaches are expanding the conceptual framework of transplant genetics beyond individual variants and HLA matching. Current evidence provides stronger support for biological relevance than clinical utility. Larger, diverse, and deeply phenotyped donor-recipient cohorts are needed to determine whether genome-wide genetic measures can improve posttransplant risk prediction and donor-recipient matching.

## INTRODUCTION

Organ transplantation creates a unique genetic setting in which phenotypes are shaped by two distinct genomes. The recipient retains their germline genome, whereas the transplanted organ introduces the donor genome. Genetic effects may therefore arise from the recipient, the donor organ, or genetic differences and interactions between them [1^▪^,2^▪^]. In transplantation, the key question is consequently not only whether a genetic variant is associated with an outcome, but also in which genome its biological effect resides and whether that effect depends on the specific donor-recipient combination.

Genome-wide approaches offer several opportunities to address this complexity. Genome-wide association studies (GWAS) identify associations between genetic variants and phenotypes in a population without restricting to prespecified candidate genes. Such an approach is particularly relevant to posttransplant outcomes, which are typically complex traits shaped by combined effects of genetic, immunological and clinical factors [1^▪^]. The variants most commonly tested in GWAS are single-nucleotide polymorphisms, but sequence variations or copy-number variants may also be tested [3^▪^]. Many common traits are highly polygenic, with numerous variants of small effect collectively contributing to a phenotype rather than a single variant exerting a large effect. The combined contribution of these variants can be captured in a polygenic risk score (PRS), in which risk alleles are weighted according to effect sizes estimated from GWAS to provide a measure of genetic predisposition to a trait or disease (Fig. 1) [3^▪^]. Together, these approaches extend genetic analyses beyond individual candidate variants towards broader measures of inherited susceptibility, graft-intrinsic genetic risk, and donor-recipient compatibility [1^▪^]. 

**Box 1:**
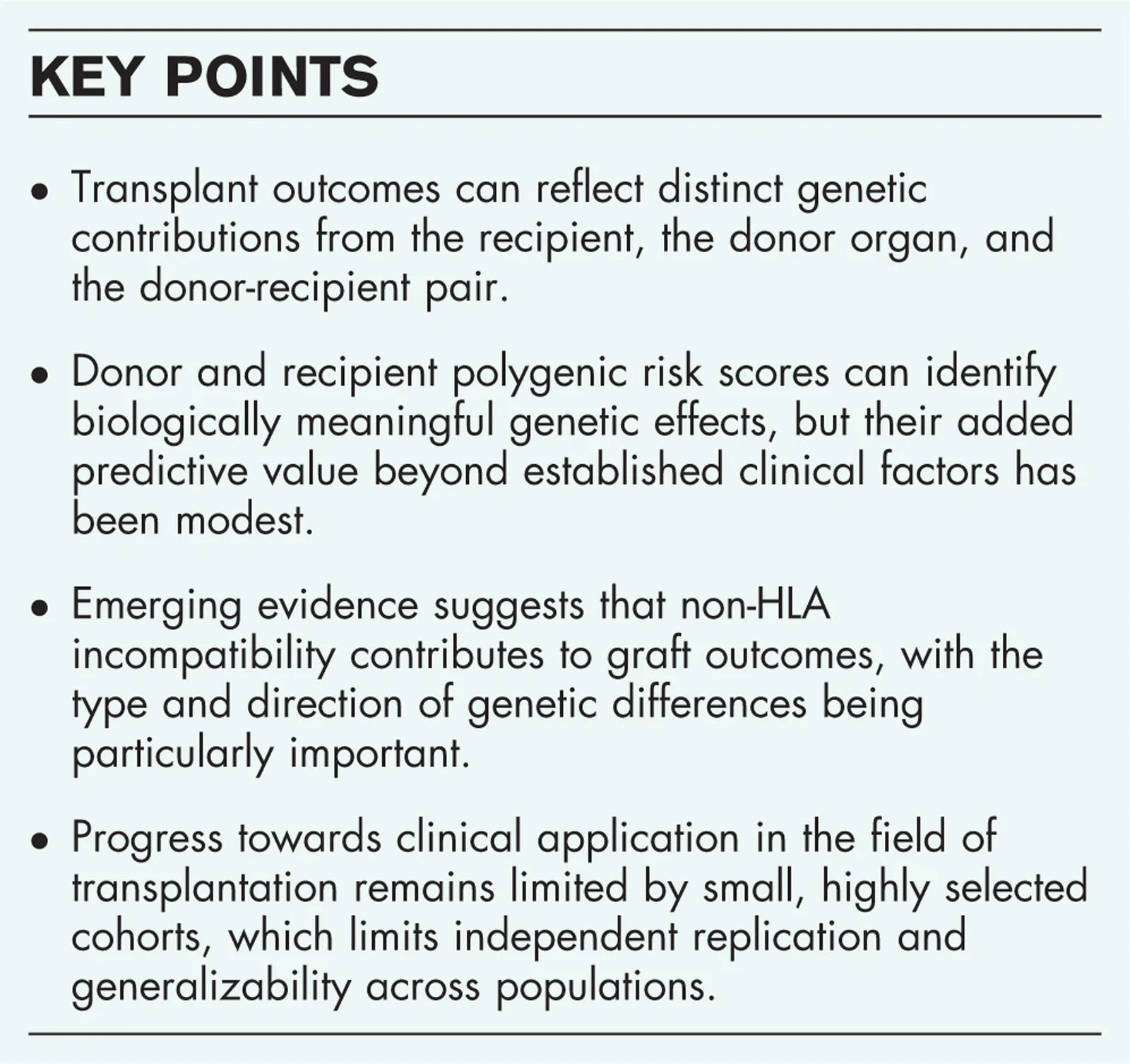
no caption available

**FIGURE 1:**
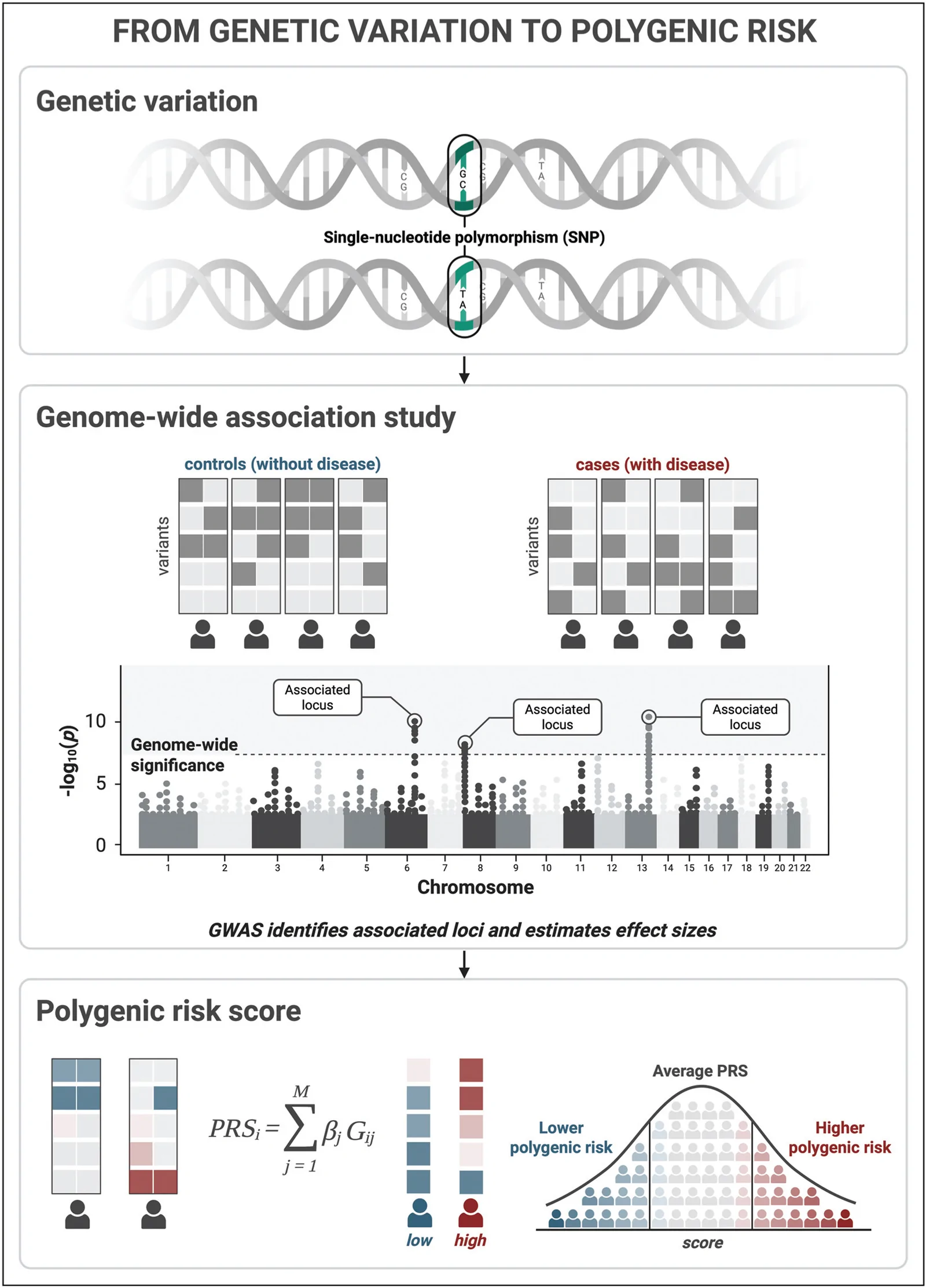
Schematic overview of genetic variation, genome wide association studies, and polygenic risk scores. Single-nucleotide polymorphisms (SNPs) represent genetic variation between individuals. In a genome-wide association study (GWAS), allele frequencies are compared between controls and cases. Thereby, genetic variations are tested for association with disease. Manhattan plots are used to visualize association signals across the genome. The dashed line indicates the threshold for genome-wide significance and highlighted peaks represent associated loci. The polygenic risk score (PRS) is calculated by combining the variant effect estimates with the number of effect alleles. The resulting PRS is the relative genetic predisposition. Lower and higher scores indicate a lower and higher polygenic risk, respectively. Blue and red in the PRS panel illustrate risk-decreasing and risk-increasing contributions. Darker shades indicate larger effect magnitudes. Created in BioRender. Stimmeder, S. (2026). https://BioRender.com/bpwng5e.

In this narrative review, we summarize developments published between 1 January 2024 and 15 August 2026 on genome-wide and polygenic analyses across heart, kidney, liver and lung transplantation.

## GENETIC EFFECTS ACROSS DONOR AND RECIPIENT GENOMES

Transplantation does not eliminate inherited susceptibility in the recipient, but places it within a new genomic context. Recipient genetic variation may continue to influence systemic processes and posttransplant outcomes, whereas donor genetic variation introduces a new genetic background through the transplanted graft [1^▪^]. As an extreme example, liver transplantation can completely correct or cure the underlying genetic and enzymatic defects of several inherited metabolic liver diseases [4]. The scope of this narrative review is, however, restricted to contemporary observational studies examining genetic variation in donor grafts and transplant recipients and its association with posttransplant outcomes.

The first GWAS of chronic lung allograft dysfunction illustrates how the relative contribution of donor and recipient genetics may differ across posttransplant phenotypes. In 392 lung transplant pairs, donor, recipient, and donor-recipient mismatch effects were analyzed separately. Two recipient loci, *PLXDC2* and *ZNF518A/BLNK*, were associated with chronic allograft dysfunction, whereas no significant donor or mismatch associations outside the human leukocyte antigen (HLA) region were identified. Although both recipient signals met the false-discovery threshold, neither reached genome-wide significance and should therefore be considered candidate loci requiring replication. Regulatory annotation and single-cell expression analyses supported a potential role for *PLXDC2 *[5]. Although these analyses move beyond statistical association toward biological mechanisms, regulatory associations remain context-dependent and cannot establish causality [5,6].

Other posttransplant phenotypes may instead be more strongly influenced by genetic variation carried within the graft. This is biologically plausible because genetic regulation of gene expression is highly tissue-dependent, with substantial variation in regulatory effects across human tissues [6]. Transplantation may therefore provide a unique setting in which tissue-intrinsic genetic effects of the donor organ can be distinguished from systemic effects of the recipient genome.

Posttransplant steatotic liver disease (SLD) provides an example. *PNPLA3* rs738409 is among the strongest established genetic determinants of SLD in the general population. After liver transplantation, however, posttransplant SLD was associated predominantly with the donor rather than the recipient genotype. Donor *HSD17B13* rs72613567-TA was likewise associated with posttransplant SLD, whereas distinct recipient variants, including *UCP2* rs695366-G, contributed independently. These findings suggest that some genetic effects are transferred with the transplanted organ, while others continue to act through the recipient [7].

A related pattern has been observed in kidney transplantation, where donor *CPB2* rs3742264 was associated with a lower risk of graft loss, whereas the corresponding recipient genotype was not. This donor-specific association may reflect local proCPB2 production within the transplanted kidney. However, rs3742264 is unlikely to be the causal variant [8]. Genetic variants that are located close together on the same chromosome are often correlated because recombination may not separate them across generations. Variants that are generally inherited together are said to be in high linkage disequilibrium, which reflects a nonrandom association between alleles at different loci. Consequently, a variant identified in an association study does not necessarily reflect the biological effect itself, but may instead act as a marker of another nearby variant which is strongly correlated [9]. In this setting, the association with rs3742264 may therefore reflect a functional *CPB2* variant in linkage disequilibrium with it [8].

This separation of donor and recipient effects also extends to polygenic susceptibility. In 1115 liver donor-recipient pairs, PRSs for type 2 diabetes, insulin secretion and insulin sensitivity were evaluated. Recipient genetic risk was predominantly related to insulin secretion, whereas donor-liver genetic effects were related to insulin sensitivity. Among recipients with low inherited type 2 diabetes risk, receiving a liver with a high donor type 2 diabetes PRS was associated with increased risk of new-onset diabetes after transplant [10].

## FROM GENETIC ASSOCIATION TO POLYGENIC RISK

Polygenic risk can be assessed in several ways in transplantation. A conventional recipient PRS estimates inherited susceptibility within the recipient, whereas a donor PRS applies the same principle to the genetic background carried by the graft. PRSs derived from both genomes reflect the possibility that a posttransplant phenotype contains independent contributions from donor and recipient. Scores constructed from genetic differences between donor and recipient are, however, conceptually distinct. They characterize the donor-recipient pair and are therefore better considered measures of genetic incompatibility or mismatch [3^▪^,^11^]. Together, these approaches offer complementary information, with donor and recipient PRSs capturing inherited susceptibility within each genome and mismatch-based scores capturing genetic differences specific to the donor-recipient pair. Recent PRS studies suggest that both may be biologically informative, while also highlighting that biological association does not necessarily translate into clinically relevant prediction.

One application of donor PRSs has been to test whether inherited susceptibility associated with the donor's cause of death remains informative after transplantation. Kidney donors who died from stroke carried a higher polygenic burden for hypertension and intracranial aneurysm than living donors and healthy controls. A higher donor hypertension PRS was subsequently associated with poorer graft survival, and donor hypertension and intracranial aneurysm PRSs were associated with lower recipient estimated glomerular filtration rate (eGFR) [12]. These findings are biologically plausible because vascular genetic risk may affect kidney function before procurement, suggesting that donor PRSs could capture inherited aspects of organ quality relevant to posttransplant outcomes. However, much of this information may already be reflected in the donor phenotype, including age, comorbidities and cause of death, potentially limiting the additional predictive value of PRS in this setting.

A subsequent analysis extended these insights by modelling PRSs in donors and recipients across 6060 transplant pairs, and similarly identified associations between donor and recipient polygenic burden and posttransplant kidney function. However, clinical variables explained approximately 25% of the variation in 1-year eGFR and 13% at 5 years, whereas inclusion of all PRSs increased the explained variance by only approximately 1%. No PRS showed an independent associated with graft failure in multivariable analysis [13]. Similarly, in posttransplant SLD, adding a five-variant genetic score to clinical predictors increased the area under the receiver-operating characteristic curve only marginally [7]. These findings demonstrate that donor and recipient polygenic effects can be statistically detectable, but may add little information beyond established clinical characteristics when the PRS-associated phenotype is closely related to clinical determinants of graft quality.

Most PRSs evaluated in transplantation are derived from GWAS performed in nontransplanted populations. Although these large cohorts offer substantially greater statistical power than transplant-specific studies, this advantage may come at the expense of generalizability. This becomes particularly relevant when scores developed in the general population are transferred to a setting that is shaped by transplantation itself, immunosuppression, and competing posttransplant clinical factors. The relevant question is therefore not simply whether a PRS remains statistically associated with an outcome, but whether the phenotype captured by the underlying GWAS remains sufficiently comparable after transplantation.

This distinction became apparent in studies focusing on the occurrence of posttransplant malignancies. A pan-cancer PRS was associated with cancer in the general population and showed an association with earlier onset of cancer among kidney recipients transplanted before 40  years of age [14]. However, the association did not remain significant after correction for multiple-testing and was not reproduced in liver and lung transplant recipients. The limited external validity may partly reflect the heterogeneous phenotype represented by a pan-cancer score, particularly because transplantation and immunosuppression substantially alter the incidence of different malignancies [14]. By contrast, a phenotype-specific PRS for actinic keratosis showed more consistent associations across transplant recipient cohorts. Each standard-deviation increase in the score was associated with up to approximately 2.5-fold higher odds of developing at least one lesion [15]. Although these studies differ in design and cannot be directly compared, their findings suggests that phenotype specificity is important and that a score developed for a relatively homogeneous phenotype may preserve more of its genetic signal after transplantation than a score that aggregates susceptibility across biologically heterogeneous outcomes.

## GENOME-WIDE DONOR-RECIPIENT INCOMPATIBILITY BEYOND HLA

The most distinctive genetic feature in transplantation may reside not in either genome separately, but in the difference between them. Although HLA provides the established model for such incompatibility, recent studies have examined whether functional variation outside the HLA region contributes to rejection and graft loss [1^▪^].

In kidney transplantation, a genome-wide scan of donor-recipient genetic mismatch in relation to acute rejection was performed in 784 living donor-recipient pairs, the majority of whom were biologically related [16]. In this cohort, 1307 genetic variants outside the HLA region were used to construct a genetic risk score, which was subsequently associated with acute rejection in an independent cohort of 352 kidney donor-recipient pairs [16].

Other studies have aimed to increase the power to detect relevant predictive genetic variants by prioritizing those with greater functional relevance. Using such a prioritization approach, a genome-wide meta-analysis of 1846 liver donor-recipient pairs found that greater functional non-HLA mismatch was associated with acute cellular rejection. Associations were also observed when the analysis was restricted to variants in transmembrane or secreted proteins, which may be particularly accessible to immune recognition. These associations remained significant after adjustment for imputed HLA mismatch [17]. A kidney transplant study similarly found that non-HLA mismatch across nonsynonymous variants in transmembrane or secreted proteins was associated with graft loss. Although this measure was uncorrelated with HLA eplet mismatch, combining HLA and non-HLA mismatch strengthened the association with graft survival [18]. These findings suggest that non-HLA variation may capture dimensions of donor-recipient compatibility that are not represented by conventional HLA matching.

Recent studies suggest that the biological relevance of non-HLA mismatch depends not simply on the number of genetic differences, but also on their type and direction. Structural variation provides a particularly informative example because a directional mismatch may expose the recipient to a gene product that is absent or expressed at substantially lower levels in their own tissues. If a recipient is homozygous for a gene deletion while the donor expresses the corresponding protein, transplantation may introduce a protein antigen to which the recipient has limited preexisting immune tolerance. This principle has been described as a genomic collision. At the *LIMS1* locus in kidney transplantation, a deletion-associated recipient genotype combined with a nondeletion donor genotype was associated with the development of anti-LIMS1 antibodies and an increased risk of rejection [19]. The importance of mismatch type and direction has also been observed in liver transplantation. Selected deletion mismatches were associated with graft rejection, whereas broader missense mismatch showed a less consistent association [20]. Together, these findings suggest that the biological relevance of non-HLA mismatch may depend less on genome-wide genetic distance than on which proteins are involved and where they are expressed.

This increasing focus on specific functional mismatches also complicates their interpretation, because an association attributed to donor-recipient incompatibility may instead reflect a direct effect of the donor or recipient genotype. In liver transplantation, homozygous *CFHR3/CFHR1* deletion in either the donor or recipient was associated with increased rejection risk, with the greatest risk when deletion was present in both. Therefore, if donor and recipient genotypes independently influence the outcome, an observed association between donor-recipient mismatch and rejection may not represent alloimmune incompatibility at all [21].

## METHODOLOGICAL AND TRANSLATIONAL CHALLENGES

Despite recent progress, transplant genomic studies remain constrained by relatively small sample sizes, partly because paired donor-recipient data, detailed phenotyping and longitudinal follow-up data are difficult to assemble. This limits statistical power and makes independent replication uncommon. Consequently, several studies rely on overlapping multicenter cohorts, and therefore the number of published associations exceeds the number of fully independent populations in which they have been evaluated [5,12,13].

Transplant cohorts are also highly selected rather than representative samples of the general population. Recipient genomes are preselected through diseases that have progressed to the point of requiring transplantation, whereas deceased-donor genomes enter the transplant pool through distinct clinical and biological pathways. This selection shapes the genetic composition of both donors and recipients, resulting in a polygenic risk distribution that differs from the general population. For example, among recipients transplanted for metabolic dysfunction-associated steatohepatitis, the frequency of the *PNPLA3* rs738409-G allele was enriched, whereas protective *HSD17B13* alleles were less frequent than in the reference population [7]. Selection is also apparent on the donor side. Donors who died from stroke carried greater polygenic burden for hypertension and intracranial aneurysm, whereas living donors had a polygenic burden comparable to healthy controls [12].

Generalizability across populations poses a further challenge. Most available cohorts are predominantly of European ancestry, limiting the applicability of PRSs and genome-wide mismatch measures across populations. A recent kidney transplant study identified four linked missense variants in *LILRB3* that were substantially more frequent among African American recipients than European recipients, with frequencies of 14.9% compared to 0.9%, respectively [22]. This illustrates how the frequency of potentially relevant genetic variants can markedly differ across ancestry groups. However, such differences are not limited to comparisons between broad ancestry groups, and may also occur within populations of similar ancestry. At the *CFH-CFHR* locus, the relationship between *CFHR3-CFHR1* markers and the underlying haplotypes differed between Dutch and U.S. cohorts, despite both populations being broadly classified as of European ancestry [21]. This shows that a tagging variant that captures a deletion or haplotype in one population may not represent the same underlying genetic exposure in another.

## CONCLUSION

Recent studies have expanded transplant genetics beyond individual variants towards genome-wide measures of risk arising from the recipient, the donor organ and the genetic relationship between them. Recipient and donor PRSs can capture biologically distinct contributions to posttransplant phenotypes, although their predictive value beyond established clinical factors has generally been modest. Emerging evidence suggests that non-HLA incompatibility extends beyond conventional HLA matching, and that the type and direction of genetic differences may be particularly important.

However, small and highly selected cohorts, limited independent replication, and restricted population diversity continue to constrain interpretation and generalizability. The field therefore currently provides stronger evidence for the biological relevance of genome-wide genetic variation than for its clinical utility. Larger, more diverse, and more deeply phenotyped cohorts will be necessary to determine whether genomic measures can meaningfully improve posttransplant risk assessment and, ultimately, donor-recipient matching.

## Acknowledgements

*None.*

### Financial support and sponsorship

*None.*

### Conflicts of interest

*V.E.M. has received institutional research grants from the Dutch Research Council (NWO; grant #09150161810030 and #09120242410037), from the Dutch Ministry of Economic Affairs (Health~Holland Public Private Partnership grant #PPP-2019-024 and #PPP-2024-010), and from the Dutch Society for Gastroenterology (NVGE #01-2021), all outside the submitted work, and has received speaker's honoraria paid to the institution by Astellas, Chiesi, and XVIVO. The other authors have nothing to disclose.*

## REFERENCES AND RECOMMENDED READING

Papers of particular interest, published within the annual period of review, have been highlighted as:

▪ of special interest
▪▪ of outstanding interest
